# A Case of Artificial Anus Following Herniotomy
1Read before the Bristol Medico-Chirurgical Society, March 13th, 1901.


**Published:** 1901-06

**Authors:** A. W. Prichard

**Affiliations:** Senior Surgeon to the Bristol Royal Infirmary.


### A CASE OF ARTIFICIAL ANUS FOLLOWING HERNIOTOMY.[1]

BY

A. W. PRICHARD,

*Senior Surgeon to the Bristol Royal Infirmary.*

---

THE following is an account of the difficulties I had in a case of strangulated hernia, and the various methods that were adopted to cure the artificial anus that necessarily resulted from the herniotomy:—

On November 24th, 1900, a woman came under my care at the Infirmary with right femoral hernia that had been strangulated for a week. She had had much poulticing and much taxis, and the symptoms of obstruction were complete. The skin was red and œdematous all round the tumour, and her general condition was grave in the extreme.

On cutting through the skin, fæcal matter exuded in more than one place, and the intestine was found to be sloughy and quite adherent at the stricture, and the patient was too bad to undergo a prolonged operation. I divided the stricture and packed it with gauze, and opened the knuckle of intestine freely.

A fortnight later sloughs and suppuration had disappeared, and the two ends of bowel were evident in the groin, surrounded by much soreness from the acrid nature of the fæcal discharge. To try and remedy this and to keep the patient nourished, the opening in the bowel being not very far from the pylorus, I introduced a glass tube bent upon itself, the ends encased in india-rubber tubing, and it was curious to see the contents of the alimentary canal passing through the tube. This did not prevent leakage, and I could not press it down enough so as to effectually alter the spur.

On December 18th I made an attempt to close the anus by an extra-peritoneal operation, but abandoned it, as I could not get enough

[1] Read before the Bristol Medico-Chirurgical Society, March 13th, 1901.

tissue free to bring across the large opening, and because the sac on the outer side was apparently incorporated with the femoral vein.

On January 15th, after due deliberation, I short-circuited the gut by establishing a lateral anastomosis after Halsted's method between the two pieces of intestine that were fixed in the groin. The abdomen was opened in the middle line, and the two columns of intestine being recognised, by the help of a colleague's fingers passed through the artificial anus, I drew the part wanted out through the wound and sutured them together by continuous silk suture. A row of Halsted's sutures were then passed, but not drawn together till after the apertures in the adjacent side of the gut were made. I made these and seamed together the mucous and peritoneal coats, which effectually stopped bleeding and rendered the apposition easy. The extremities were strengthened by a few more Halsted's sutures. I did nothing to the part of the gut between the anastomosis and the artificial anus. The result at first was most encouraging, the bowels acting in the ordinary way and the amount coming from the artificial anus proportionately diminishing. I left matters alone, thinking that the herniated part might shrink; but, instead of that, the anastomosis in a short time did not work properly, and in three weeks' time closed up so that I could get no communication between the columns on testing by injections of coloured solutions.

I now fell back upon my *dernier ressort*—a plan I wished to try before the lateral anastomosis, only my colleagues persuaded me otherwise—that was the use of Dupuytren's enterotome. I passed the two blades up to the hilt, locked them and screwed them up, and the second day screwed them nearly as far as it was possible. The two blades seemed to be nearly in contact on the fifth day, the outside part being screwed as tightly as possible; but it was not till the twelfth day that the instrument fell out, my forefinger passing through the cleft with the greatest ease. There had been no pain or discomfort from the wearing of the instrument. Three days after the enterotome came away, I closed the anus in the following manner:—Much of the exuberant mucous and sub-mucous coats was cut off. Then the outer coats were dissected from the skin tissues until there was sufficient to meet in the middle line, a small line of neighbouring skin being included. The mucous coat that was left was then stitched together in the middle line by catgut, forming a mucous roof to the new canal. The muscular and serous coats were sutured over the mucous roof by Halsted's silk sutures, and then the skin, the subcutaneous areolar tissue having been freed, was slid from each side and sewn together in the middle by a row of silkworm gut stitches. An iodoform gauze plug was left in at each end for drainage.

This was done on the 4th of March, and the result has been good. Glycerine enemata from three days after the operation brought about a natural result, and the whole of the wound healed. A stitch abscess formed, discharging some fœtid pus; but the patient is now practically well.

